# Estimated Indirect Cost Savings of Using Telehealth Among Nonelderly Patients With Cancer

**DOI:** 10.1001/jamanetworkopen.2022.50211

**Published:** 2023-01-10

**Authors:** Krupal B. Patel, Kea Turner, Amir Alishahi Tabriz, Brian D. Gonzalez, Laura B. Oswald, Oliver T. Nguyen, Young-Rock Hong, Heather S. L. Jim, Anthony C. Nichols, Xuefeng Wang, Edmondo Robinson, Cristina Naso, Philippe E. Spiess

**Affiliations:** 1 Department of Head and Neck and Endocrine Oncology, Moffitt Cancer Center, Tampa, Florida; 2 Department of Otolaryngology, Head and Neck Surgery, University of South Florida, Tampa; 3 Department of Health Outcomes and Behavior, Moffitt Cancer Center, Tampa, Florida; 4 Department of Health Services Research, Management and Policy, College of Public Health and Health Professions, University of Florida, Gainesville; 5 Department of Otolaryngology, Head and Neck Surgery, Western University, London, Ontario, Canada; 6 Department of Biostatistics and Bioinformatics, Moffitt Cancer Center, Tampa, Florida; 7 Department of Internal and Hospital Medicine, Moffitt Cancer Center, Tampa, Florida; 8 Center for Digital Health, Moffitt Cancer Center, Tampa, Florida; 9 Virtual Health Program, Moffitt Cancer Center, Tampa, Florida; 10 Department of Genitourinary Oncology, Moffitt Cancer Center, Tampa, Florida

## Abstract

**Importance:**

Patients with cancer typically have greater financial hardships and time costs than individuals without cancer. The COVID-19 pandemic has exacerbated this, while posing substantial challenges to delivering cancer care and resulting in important changes in care-delivery models, including the rapid adoption of telehealth.

**Objective:**

To estimate patient travel, time, and cost savings associated with telehealth for cancer care delivery.

**Design, Setting, and Participants:**

An economic evaluation of cost savings from completed telehealth visits from April 1, 2020, to June 30, 2021, in a single-institution National Cancer Institute–Designated Comprehensive Cancer Center. All patients aged 18 to 65 years who completed telehealth visits within the designated time frame and had a Florida mailing address documented in their electronic medical record were included in the study cohort. Data were analyzed from April 2020 to June 2021.

**Main Outcomes and Measures:**

The main outcome was estimated patient cost savings from telehealth, which included 2 components: costs of travel (defined as roundtrip distance saved from car travel) and potential loss of productivity due to the medical visit (defined as loss of income from roundtrip travel plus loss of income from in-person clinic visits). Two different models with a combination of 2 different mileage rates ($0.56 and $0.82 per mile) and census tract–level median hourly wages were used.

**Results:**

The study included 25 496 telehealth visits with 11 688 patients. There were 4525 (3795 patients) new or established visits and 20 971 (10 049 patients) follow-up visits. Median (IQR) age was 55.0 (46.0-61.0) years among the telehealth visits, with 15 663 visits (61.4%) by women and 18 360 visits (72.0%) by Hispanic non-White patients. According to cost models, the estimated mean (SD) total cost savings ranged from $147.4 ($120.1) at $0.56/mile to $186.1 ($156.9) at $0.82/mile. For new or established visits, the mean (SD) total cost savings per visit ranged from $176.6 ($136.3) at $0.56/mile to $222.8 ($177.4) at $0.82/mile, and for follow-up visits, the mean (SD) total cost savings per visit was $141.1 ($115.3) at $0.56/mile to $178.1 ($150.9) at $0.82/mile.

**Conclusions and Relevance:**

In this economic evaluation, telehealth was associated with savings in patients time and travel costs, which may reduce the financial toxicity of cancer care. Expansion of telehealth oncology services may be an effective strategy to reduce the financial burden among patients with cancer.

## Key Points

**Question:**

What are the estimated cost savings of using telehealth among patients with cancer?

**Findings:**

This economic evaluation of cost savings from completed telehealth appointments included 11 688 patients younger than 65 years, with 25 496 telehealth visits at a National Cancer Institute–Designated Comprehensive Cancer Center from April 1, 2020, to June 30, 2021. According to cost models, the estimated mean total cost savings ranged from $147.4 to $186.1 per visit.

**Meaning:**

These findings suggest that telehealth saves time, travel, and money for patients, which could improve care delivery and may reduce the financial toxicity of cancer care.

## Teaser

This economic evaluation estimates the indirect cost savings of using telehealth among nonelderly patients with cancer.

## Introduction

Financial toxicity includes both objective financial burden (ie, costs) and subjective financial distress.^1,2^ Costs of cancer care include: direct cost of care (cost sharing through higher deductibles, copayments, coinsurance, and even entire cost of care for uninsured patients) and indirect costs of care (lost productivity and cost of driving to and from appointments).^1,3^ Patients with cancer have greater time-based costs than those without cancer (eg, time spent traveling back and forth to appointments and time spent receiving medical care).^4,5,6^ Strategies are needed to reduce the direct and indirect costs of cancer care delivery.

The rapid adoption of telehealth during the COVID-19 pandemic has allowed patients to receive care in a location that is convenient for them, which may reduce the costs of cancer care. To date, there has been limited research regarding the cost savings of telehealth among patients with cancer. The COVID-19 pandemic is providing a unique opportunity to estimate the potential cost savings of telehealth in oncology care.^7^ Although it is well established that patients with cancer experience substantial financial toxicity, few studies have explored the indirect costs that they face. Thus, this study focused specifically on an oncologic population from a comprehensive cancer center with a substantially large sample size to estimate the indirect cost savings (driving costs and lost productivity) from telehealth visits.

## Methods

This was an economic evaluation estimating cost savings from completed telemedicine visits at Moffitt Cancer Center (MCC), the only National Cancer Institute (NCI) –Designated Comprehensive Cancer Center in Florida. Data from telehealth visits were collected from April 1, 2020, to June 30, 2021. All patients aged between 18 and 65 years who completed telehealth visits within the designated time frame and had a Florida mailing address documented in their electronic medical record were included in the study cohort. All patients were offered telehealth if deemed appropriate by the clinical team. Telehealth visits were not offered to patients who needed physical examinations beyond what can be assessed during a telehealth visit. Patients who presented in person for chemotherapy infusion and/or radiation treatment were excluded from the analysis. This study was exempt from MCC institutional review board approval with a waiver of informed consent from patients because the study was deemed low risk. This study used the Consolidated Health Economic Evaluation Reporting Standards (CHEERS) reporting guideline.^8^

Due to the COVID-19 pandemic, implementation of telehealth at MCC was accelerated in March 2020. Telehealth was defined as care delivered through a videoconferencing platform in real time. Starting in April 2020, MCC instituted videoconferencing for their telehealth visits. Patient visits were defined as new, established, or follow-up. New patient visits were patients not having received any previous medical care from MCC; established patient visits had received care at MCC previously but were referred to a new subspecialty for consultation; and follow-up patient visits were seen at MCC for follow-up care by clinicians in the same subspecialty they had previously received care from.

### Statistical Analysis

**Figure 1.**
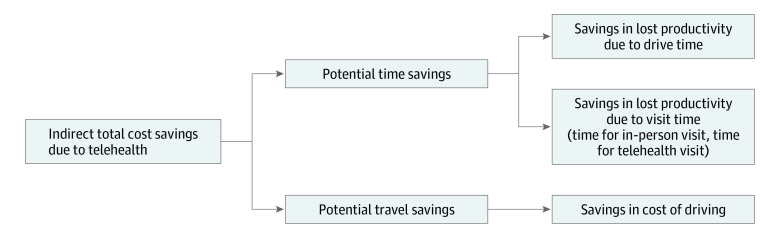
Graphical Representation of Calculation of Estimated Indirect Total Cost Savings Due to Telehealth Potential time savings was defined as roundtrip time savings arising from the use of telehealth, calculated as the difference between the time required to travel from the patient’s home address to in-person consultation at the Moffitt Cancer Center (MCC) plus the in-person consultation time vs the time required to attend a telehealth visit from home (ie, time savings = roundtrip drive time + [time for in-person consultation − time for telehealth visit]). Potential travel savings was defined as roundtrip distance savings arising from the use of telemedicine, calculated as the distance the patient would have traveled for an in-person consultation at the MCC.

We assessed patient time, travel, and indirect cost savings from using telehealth for cancer care delivery (Figure 1). Analyses were guided by the framework recommended by Sanders and colleagues^9^ for assessing the time and transportation costs of patients. Time savings were calculated as the difference between the roundtrip time required to travel from each patient’s home address to an in-person consultation at MCC, plus in-person consultation time vs the time required to attend a telehealth visit from home (ie, time savings = roundtrip drive time + [time for in-person consultation – time for telehealth visit]). Travel savings were calculated as the roundtrip driving distance in miles from each patient’s home address to an in-person consultation at MCC. Indirect cost savings were calculated as the roundtrip costs associated with traveling from each patient’s home address to an in-person consultation at MCC. This included 2 components: the costs of travel and the potential loss of productivity due to the medical visit.

The American Community Survey (ACS)^10^ was used to determine census tract–level data for hourly median income per year. The census tract income data were then matched to the patient’s address. This analysis focused on patients younger than 65 years, because these patients were more likely to be employed full time than those aged 65 years or older.

Two different models were generated with a combination of 2 different mileage rates and hourly wage rates determined via ACS census tract level data. Driving distance traveled in miles was calculated in October 2021 by Buxton Company,^11^ an analytics organization that uses Alteryx’s^12^ analytic platform to provide geospatial data. Briefly, the locations are geocoded, and distance is calculated between the 2 geocoded locations by finding the route that results in the least amount of drive time between the 2 locations.

Calculations for different models were conducted using R (R Project for Statistical Computing).^13^ Details are available in the eMethods in Supplement 1. Data were analyzed from April 2020 to June 2021.

## Results

**Figure 2.**
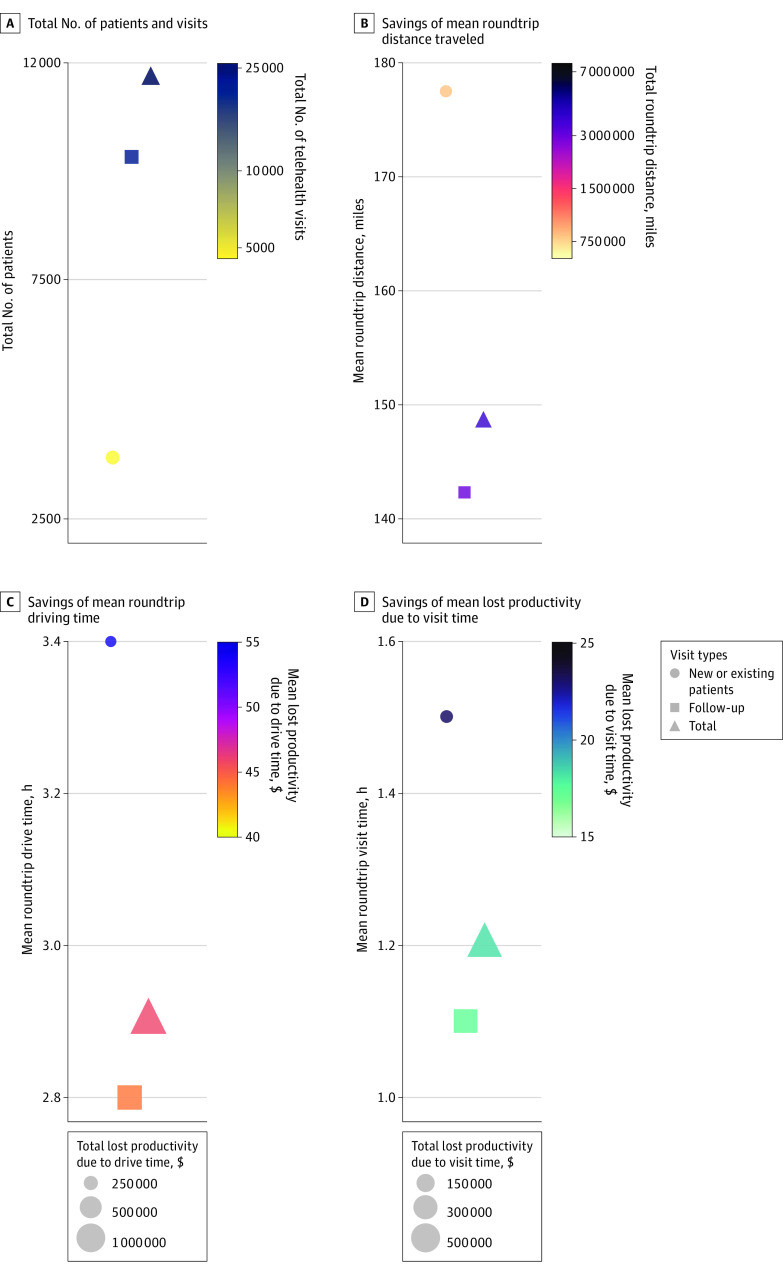
Estimated Total Number of Visits, Roundtrip Drive Time, Roundtrip Distance, Total and Mean Lost Productivity Due to Drive Time, and Total and Mean Lost Productivity Due to Visit Time A, Total number of patients and visits. B, Savings of mean roundtrip distance traveled. Color represents the total roundtrip distance saved for all patients. C, Savings of mean roundtrip driving time. Color represents the mean lost productivity due to driving time; the size of the marker represents total lost productivity due to drive time. D, Savings of mean lost productivity due to visit time. Color represents the mean lost productivity due to visit time; the size of the marker represents total lost productivity due to visit time.

A total of 25 496 telehealth visits for 11 688 patients were conducted for patients aged between 18 and 65 years during the study period. There were 4525 (3795 patients) new or established visits and 20 971 (10 049 patients) follow-up visits (Figure 2A). The eTable in Supplement 1 highlights the demographics of the telehealth visits. Median (IQR) age was 55.0 years (46.0-61.0) among the telehealth visits, with 15 663 visits (61.4%) being women, 18 443 visits (72.3%) having private insurance, and 18 360 (72.0%) visits by White non-Hispanic individuals. In travel, an estimated 3 789 963 roundtrip miles (804 969 for new or established visits and 2 984 994 for follow-up visits) were saved, equating to 75 055 hours (15 422 new or established visits and 59 633 for follow-up visits) of savings in total driving time. Per visit, telehealth was associated with mean (SD) savings of 148.6 (143.7) roundtrip travel miles and 2.9 (2.3) hours of roundtrip driving time (Table 1, Figure 2B and 2C). An additional 29 626 hours of in-clinic visits were saved by using telehealth with a mean (SD) savings of 1.2 (0.13) hours per visit (Figure 2D). For new or established visits, telehealth was associated with mean (SD) savings of 177.6 (161.6) roundtrip travel miles, 3.4 (2.6) hours of roundtrip driving time and 1.5 (0.0) hours of in-clinic time per visit (Figure 2B, 2C, and 2D). For follow-up visits, telehealth was associated with mean (SD) savings of 142.4 (138.8) roundtrip travel miles, 2.8 (2.3) hours of roundtrip driving time and 1.1 (0.0) hours of in-clinic time per visit (Figure 2B, 2C, and 2D).

**Table 1.** Total Number of Visits, Roundtrip Drive Time, and Roundtrip Distance of Virtual Visits Seen at Moffitt Cancer Center From April 1, 2020, to June 30, 2021

Characteristic	NP/EP, No.^a^	Follow-up, No.^a^	Total, No.
No. of patients	3795	10 049	11 688
No. of visits	4525	20 971	25 496
Total roundtrip, miles	804 969	2 984 994	3 789 963
Roundtrip, mean (SD), miles	177.6 (161.6)	142.4 (138.8)	148.6 (143.7)
Total roundtrip driving time, h	15 422	59 633	75 055
Roundtrip driving time, mean (SD), h	3.4 (2.6)	2.8 (2.3)	2.9 (2.3)
Visit time saved, h	6561	23 068	29 626
visit time saved, mean (SD), h	1.5 (0.0)	1.1 (0.0)	1.2 (0.13)

Abbreviations: EP, existing patients referred to a different subspecialty; NP, new patients.

^a^ A portion of patients will have both NP/EP and subsequent follow-up visits.

**Figure 3.**
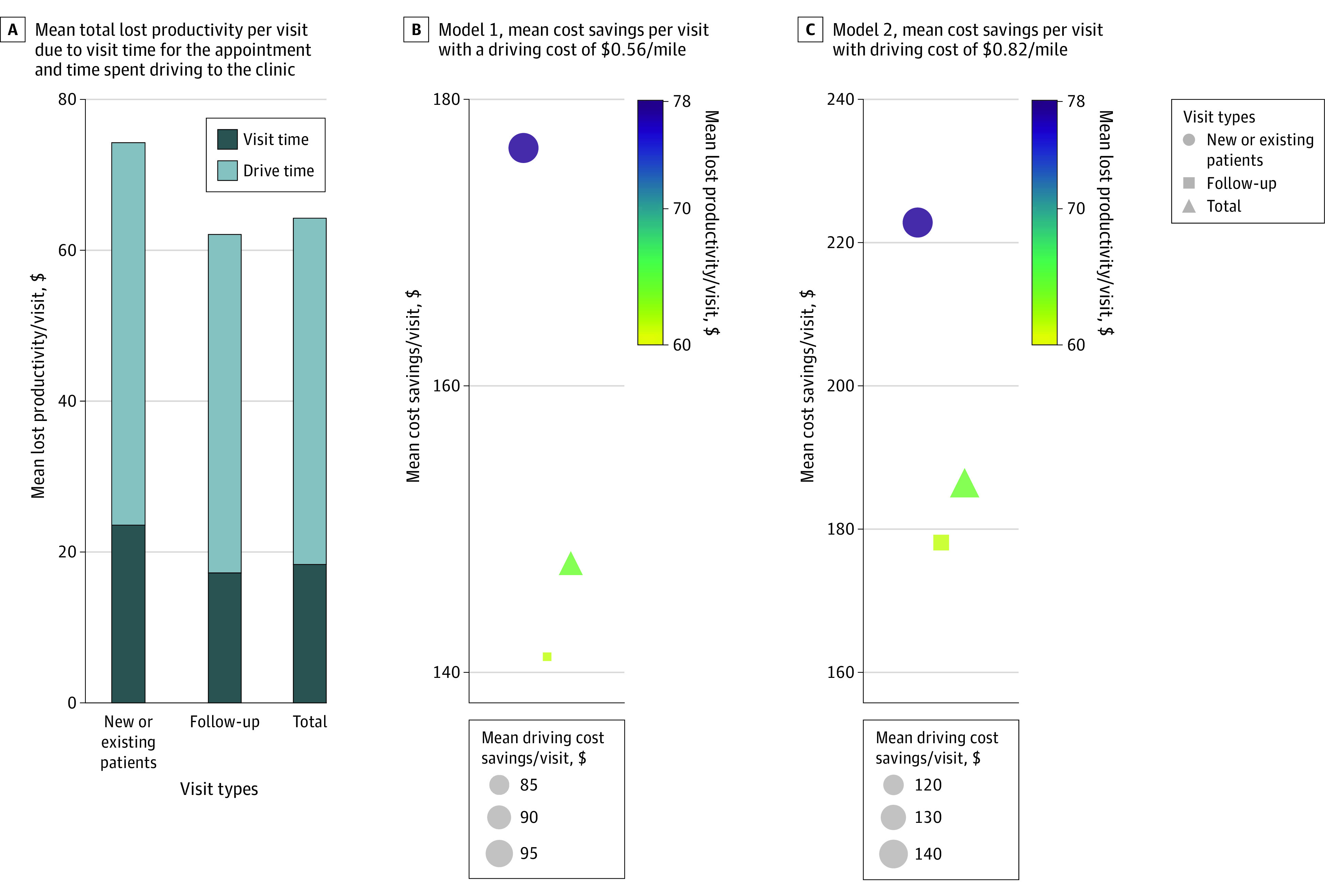
Projected Cost Savings of Virtual Visits Seen at Moffitt Cancer Center From April 1, 2020, to June 30, 2021 Two different models were used with a range of costs per mile ($0.56/mile and $0.82/mile). A, Mean total lost productivity per visit due to visit time for the appointment and time spent driving to the clinic. B, Model 1, mean cost savings per visit with a driving cost of $0.56/mile. Color represents mean savings from lost productivity per visit (due to the visit time and driving time), and the size of the marker represents mean driving cost savings/visit. C, Model 2, mean cost savings per visit with driving cost of $0.82/mile. Color represents mean savings from lost productivity per visit (due to visit time and driving time) and the size of the marker represents the mean driving cost savings per visit.

Telehealth was associated with an estimated $1 170 160 savings in lost productivity (income) due to driving time, $467 247 savings in lost productivity due to visit time, and $1 637 407 total savings in lost productivity (Table 2, Figure 2B, 2C, and 2D). For new or established visits, the following savings were noted: $245 113 savings in lost productivity due to driving time, $104 522 savings in lost productivity due to visit time, and $349 655 total savings in lost productivity. For follow-up visits, the following savings were noted: $925 027 savings in lost productivity due to driving time, $362 725 savings in lost productivity due to visit time, and $1 287 752 total savings in lost productivity. Mean (SD) savings in lost productivity per visit due to driving time were $45.9 (41.5) per visit overall, and $54.1 (47.9) for new or established visits and $44.1 (39.7) for follow-up visits (Table 2, Figure 3A). Mean (SD) savings per visit in lost productivity due to visit time was $18.3 (5.9) per visit overall, and $23.1 (6.9) for new or established visits and $17.3 (5.1) for follow-up visits. Estimated mean (SD) total savings per visit from lost productivity was $64.2 (43.6) per visit overall, $77.2 (50.6) for new or established visits, and $61.4 (41.4) for follow-up visits. Total driving-cost savings ranged from $2 122 379 at $0.56/mile (Figure 3B) to $3 107 777 at $0.82/mile (Figure 3C). For new or established visits, total driving-cost savings were $450 782 at $0.56/mile to $660 074 at $0.82/mile, while for follow-up visits, total driving-cost savings were $1 671 597 at $0.56/mile to $2 447 695 at $0.82/mile. According to cost models, the mean (SD) driving cost savings per visit ranged from $83.2 ($80.5) at $0.56/mile to $122.0 ($118.0) at $0.82/mile (Table 2, Figure 3B and 3D). For new or established visits, the mean (SD) driving cost savings per visit ranged from $99.6 ($90.5) at $0.56/mile to $146.0 ($132.6) at $0.82/mile, and for follow-up visits, the mean (SD) cost savings per visit was $79.7 ($77.7) at $0.56/mile to $116.7 ($113.8) at $0.82/mile. According to cost models, the mean (SD) total cost savings per visit ranged from $147.4 ($120.1) at $0.56/mile to $186.1 ($156.9) at $0.82/mile (Table 2, Figure 3B and 3D). For new or established visits, the mean (SD) total cost savings per visit ranged from $176.6 ($136.3) at $0.56/mile to $222.8 ($177.4) at $0.82/mile, and for follow-up visits, the mean total cost savings per visit was $141.1 ($115.3) at $0.56/mile to $178.1 ($150.9) at $0.82/mile.

**Table 2.** Projected Cost Savings of Virtual Visits Seen at Moffitt Cancer Center From April 1, 2020, to June 30, 2021

Savings type	NP/EP, No.^a^	Follow-up, No.^a^	Total, No.
Savings in lost productivity (income) due to driving time, $	245 113	925 027	1 170 160
Savings in lost productivity per visit due to driving time, mean (SD), $	54.1 (47.9)	44.1 (39.7)	45.9 (41.5)
Savings in lost productivity due to visit time, $	104 522	362 725	467 247
Savings in lost productivity due to visit time, mean (SD), $	23.1 (6.9)	17.3 (5.1)	18.3 (5.9)
Total savings in lost productivity, $	349 655	1 287 752	1 637 407
Total savings in lost productivity per visit, mean (SD), $	77.2 (50.6)	61.4 (41.4)	64.2 (43.6)
$0.56/mile^b^			
Savings in total driving costs, $	450 782	1 671 597	2 122 379
Driving cost savings per telehealth visit, mean (SD), $	99.6 (90.5)	79.7 (77.7)	83.2 (80.5)
Total cost savings per telehealth visit, mean (SD), $	176.6 (136.3)	141.1 (115.3)	147.4 (120.1)
$0.82/mile^b^			
Savings in total driving costs, $	660 074	2 447 695	3 107 777
Driving cost savings per telehealth visit, mean (SD), $	146.0 (132.6)	116.7 (113.8)	122.0 (118.0)
Total cost savings per telehealth visit, mean (SD), $	222.8 (177.4)	178.1 (150.9)	186.1 (156.9)

Abbreviations: EP, existing patients referred to a different subspecialty; NP, new patient.

^a^ A portion of patients will have both NP/EP and subsequent follow-up visits.

^b^ Two different models were used with a range of costs per mile ($0.56/mile and $0.82/mile).

## Discussion

This economic evaluation study uses a large data set collected at an NCI-Designated Comprehensive Cancer Center to estimate patients’ savings from using telehealth. From April 1, 2020, to June 30, 2021, a total of 25 496 telehealth visits were conducted. Telehealth was associated with a total savings of 3 789 963 roundtrip travel miles, which equates to traveling 152.2 times around the earth, and a total savings of 75 055 roundtrip drive hours, which equates to 8.6 calendar years. An additional 3.4 calendar years (29 626 hours) were saved in clinic visits by using telehealth. Depending on the visit types, mean savings in lost productivity per visit due to driving time ranged from $44.1 to $54.1, mean savings in lost productivity due to visit time ranged from $17.3 to $23.1, and mean total savings in lost productivity per visit ranged from $61.4 to $77.2. Mean driving cost savings per telehealth visits ranged from $79.71 to $146.0 depending on visit type and model used. Mean total cost savings per visit ranged from $141.1 to $222.8 depending on the visit type and model used.

Some of the main arguments for implementing telehealth are to increase access to care, patient convenience, and cost savings in outpatient clinics.^14^ Telehealth may also provide an opportunity to reduce emergency department visits, readmissions, and patient mortality.^14^ As patients’ financial costs of cancer care increase, telehealth may reduce their burden of travel including costs associated with parking and lodging, and lost income from missing work.

The burden of travel has been identified as an important factor that can change access to diagnosis, treatment of cancer and participation in clinical trials.^15,16^ Transportation is a key determinant of health care access and has been identified as an important source of out-of-pocket nonmedical costs for patients receiving cancer care.^17^ Patients without adequate transportation are more likely to miss appointments and rely on emergency department care,^15^ and there is substantial variability in the estimated parking costs throughout cancer treatment.^18^ In addition, a recent study noted that the number of rural hospitals has decreased over the last decade, resulting in almost double the number of people living outside a 60-minute radius of major hospitals and longer drive times to receive care.^19^ Thus, telehealth could be beneficial among rural patients in particular.^20^

In the models previously mentioned, we did not consider the cost savings of telehealth for cancer caregivers. Caregivers for patients with cancer spend substantial time and effort to coordinate and attend appointments with patients. In 2020, 53 million individuals were caregivers, 6% of whom were caregivers for patients with cancer. The vast majority of caregivers (80%) help with transportation; 18% report high financial strain; and 45% have experienced at least 1 financial impact as a result of caregiving.^21^ Although the current study was focused on indirect cost savings from patients’ perspectives, future studies should include caregivers’ indirect cost savings as often patients and caregivers function as a unit and share expenses. Therefore, savings from telehealth would be even higher if caregivers’ savings from lost productivity were accounted for, especially when telehealth has the ability for multiple caregivers to join the same appointment from various geographical locations.

Although telehealth offers considerable cost savings to patients with cancer, it is well documented that telehealth adoption is affected by the digital divide. Factors associated with financial toxicity (eg, age, insurance, race, and education) are also associated with the digital divide.^22^ Future studies are needed to address inequities in telehealth uptake. Additionally, telehealth requires substantial infrastructure costs and investments from health systems with buy-in from administrators and clinicians to ensure high patient satisfaction.^23^

### Limitations

Our study has several limitations. This analysis was retrospectively conducted at a tertiary/quaternary referral center, and so roundtrip travel distances may be higher than usual because this is a destination center for cancer care. Our assumption of employment rates and incomes for patients younger than 65 years may vary. Additionally, a percentage of patients on active treatment or following treatment may not be fully employed given their functional status, thus affecting the savings from lost productivity. Because we were unable to accurately capture employment among older adults, patients aged over 65 years were excluded; however, future studies should examine cost savings in this population. Cost savings due to lost productivity assumed that all patients are nonsalaried and the loss due to travel time and hours of visit time could not be made up for. Therefore, the savings in this study might be considered a maximum amount of lost productivity. This study only considered telehealth visits that were completed via synchronous videoconference, and the costs of electronic devices and internet access were not considered. This study also did not assess other factors likely to affect cost savings, such as rural vs urban residences, race, education, or insurance type, all of which should be explored in future studies. Finally, further data are needed if long-term oncologic outcomes with telehealth visits are equivalent to those seen in person, which can change costs of treatment.

## Conclusions

Patients with cancer spend a substantial amount of time and money traveling to receive care. Using a large data set, we found that cancer care delivery via telehealth was associated with time, travel, and cost savings for patients with cancer, which may reduce the financial toxicity of cancer care. Future studies should explore other cost savings, such as the savings to cancer caregivers and how these vary for rural and urban patients with cancer.

## Notes

Supplement 1.
eMethods

eReferences
**eTable.** Demographics of Telehealth Visits Seen at Moffitt Cancer Center From April 1, 2020, to June 30, 2021

Supplement 2.
Data Sharing Statement
