# Supplementary material for: Estimated Indirect Cost Savings of Using Telehealth Among Nonelderly Patients With Cancer
Source: JAMA Netw Open. 2023 Jan 10;6(1):e2250211. doi: 10.1001/jamanetworkopen.2022.50211 (PMC9856804; doi:10.1001/jamanetworkopen.2022.50211)
Supplement: Supplement 1. — eMethods eReferences eTable. Demographics of Telehealth Visits Seen at Moffitt Cancer Center From April 1, 2020, to June 30, 2021 [file jamanetwopen-e2250211-s001.pdf]

Supplemental Online Content

Patel KB, Turner K, Tabriz AA, et al. Estimated indirect cost savings of using telehealth among nonelderly patients with cancer. *JAMA Netw Open*. 2023;6(1):e2250211. doi:10.1001/jamanetworkopen.2022.50211

eMethods

eReferences

eTable. Demographics of Telehealth Visits Seen at Moffitt Cancer Center From April 1, 2020, to June 30, 2021

This supplemental material has been provided by the authors to give readers additional information about their work.

© 2023 Patel KB et al. *JAMA Network Open.*

eMethods:

Study Sample: This was a retrospective cohort study of patients, 18 – 65 years of age, seen at Moffitt Cancer Center (MCC), the only NCI designated cancer center in the state of Florida. Due to COVID-19 pandemic, implementation of telehealth at MCC was accelerated in March 2020. Telehealth was defined as care delivered through a videoconferencing platform in real-time. Starting in April 2020, MCC instituted Zoom platform for their telehealth visits. To determine potential travel savings, time savings and cost savings, data for patients with Florida addresses was used for telehealth visits from April 2020 to June 30, 2021. New patients were completely new to MCC, established patients had received care at MCC previously but were referred to a new sub-specialty for consultation, and follow-up patients were being seen for follow-up care by providers within the same sub-specialty. New patient and established patient visit times were similar and thus these categories were considered same.

Data analyses

Potential indirect cost savings was defined as round-trip cost savings arising from the use of telemedicine, calculated as the difference between the travel costs associated with traveling from the patient's home address to an in-person consultation at the MCC. This included 2 components: 1) cost of travel, and 2) potential loss of productivity due to the medical visit.

Potential time savings was defined as round-trip time savings arising from the use of telehealth, calculated as the difference between the time required to travel from the patient's home address to in-person consultation at the MCC plus the in-person consultation time vs. the time required to attend a telehealth visit from home i.e. time savings = roundtrip drive time + (time for in-person consultation – time for telehealth visit). Previous study has reported an average clinic time of 84 minutes, however there was no distinction between new patients or follow up appointments.[1] To more accurately, determine the potential time savings, institutional in person and telehealth mean duration of each visit types were used. Mean duration for in person visits were determined based on clinic cycle times (patient check in to patient check out) – 96.4 minutes for new/established visits and 58.1 minutes for follow up visits. Additional 30 minutes were added to each of these in person visits for parking. Thus, a new/established visit took 126.4 minutes (96.4 + 30 minutes) for new/established visits and 88.1 minutes (58.1 + 30 minutes) for follow up visits. Mean duration for telehealth visits were determined based on clinician's join time and log out time for the visit – 39.2 minutes for new/established visits and 22.1 minutes for follow up visits. The methodology for determining travel time was based on driving distance between patient's address listed in the chart and MCC (all totals were based on round trip drive time calculations). For patients with Florida addresses, we used the respective patient address as a driving departure point and for a small minority of the patients whose addresses were not available, zip codes were used as a point of origin for travel time and distance calculation. American Census Survey (ACS)[2] was used to determine census tract level data for hourly median income per year which was divided 2080 hours to determine the hourly wage. The census tract income data was then matched to the patient's address to get more accurate representation of hourly wages.

Potential travel savings was defined as round-trip distance savings arising from the use of telemedicine, calculated as the distance the patient would have traveled for an in-person consultation at the MCC. The methodology for determining the distance was based on patient's address listed in the chart. For patients with Florida addresses, we used the respective patient address as a driving departure point and Moffitt Cancer Center (MCC) as a final destination (all totals were based on round trip calculations). For

those patients with postal office as their mailing address, zip code's centroid was used as driving departure point.

Cost of operating a vehicle was determined based on a) Internal Revenue Services' 2020 standard mileage rate of $0.56/mile[3], or b) American Automobile Association standard mileage rate of $0.82/mile[4] multiplied by distance travelled for each round trip.

Two different models were generated with a combination of two different mileage rates and hourly wage rate determined via ACS census tract level data.

Driving distance travelled in miles were calculated in October 2021 by Buxton Company (Fort Worth, TX)[5], an analytics organization that uses Alteryx's[6] analytic platform to provide geospatial data. Briefly, the locations are geocoded. Distance is calculated between the two geocoded locations by finding the route that results in the least amount of drive time between the two locations. Calculations for different models were conducted using R.[7]

© 2023 Patel KB et al. *JAMA Network Open.*

eReferences:

1. Ray KN, Chari AV, Engberg J, Bertolet M, Mehrotra A. Opportunity costs of ambulatory medical care in the United States. *Am J Manag Care*. Aug 2015;21(8):567-74.
2. American Community Survey (ACS) (2019).
3. Services. IR. IRS issues standard mileage rates for 2021. Internal Revenue Services. Accessed October 1, 2021, 2021. https://www.irs.gov/newsroom/irs-issues-standard-mileage-rates-for-2021
4. Association AA. How much does it really cost to own a new car? American Automobile Association. Accessed October 1, 2021. https://newsroom.aaa.com/wp-content/uploads/2020/12/2020-Your-Driving-Costs-Brochure-Interactive-FINAL-12-9-20.pdf
5. Buxton Company. Accessed October 15, 2021. https://www.buxtonco.com/
6. Alteryx. Accessed October 15, 2021. https://www.alteryx.com/
7. *R: A Language and Environment for Statistical Computing*. 2019. https://www.R-project.org/

© 2023 Patel KB et al. *JAMA Network Open.*

eTable 1. Demographics of Telehealth Visits Seen at Moffitt Cancer Center From April 1, 2020, to June 30, 2021

	All Patients			Patients under the age 65 yrs	
	NP/EP[a]	FU[a]	Total	NP/EP[a]	FU[a]
**Number of unique patients**	7350	19 964	23 228	3795	10 049
**Number of visits**	8743	40 586	49 329	4525	20 971
**Age (median, IQR)**	65.0 (54.0 – 73.0)	65.0 (55.0 – 73.0)	65.0 (55.0 – 73.0)	55.0 (46.0 – 61.0)	55.0 (46.0 – 61.0)
**Sex**					
Males	3805	18 817	22 622	1660	8 174
Females	4938	21769	26 707	2865	12798
**Insurance**					
Private	3731	16 392	20 123	3415	15 028
Medicare	4184	20 572	24 756	420	2824
Medicaid	338	1 808	2 146	319	1709
Others	490	1 814	2 304	371	1410
**Race/Ethnicity**					
Non-Hispanic Whites	6305	32 084	38 389	3018	15 342
Hispanic Whites	489	2378	2867	350	1677
Blacks	465	2285	2750	315	1640
Others	1484	3839	5323	842	2312
**Clinic Type**					
Anesthesia		1415	1 15		887
BMT/Malignant Hematology	607	5079	5686	245	2183
Breast	320	2205	2525	229	1482
Cutaneous/Sarcoma	232	2571	2803	118	1318
Endocrine/HN	403	3668	4071	273	2423
Genetics	1766	1075	2841	1152	686
GI Tumor	849	2846	3695	396	1284
GU Oncology	504	3979	4483	196	1467
Gyn	52	734	786	31	441
IHM/IM	246	1103	1349	120	539
Infectious Disease	63	142	205	33	69
Integrative Medicine	43	103	146	28	96
Interventional Pain	53	967	1 020	31	547
Interventional Radiology	283	257	540	119	75

© 2023 Patel KB et al. *JAMA Network Open.*

Neuro Oncology	227	1904	2131	134	1284
Rad Oncology	1128	2777	3905	478	1161
Supportive Care Medicine	496	2939	3435	305	2099
Survivorship	455	43	498	235	16
Thoracic Oncology	636	2113	2749	240	752
Others	379	4667	5046	161	2163

Abbreviations: EP, existing patients referred to a different subspecialty; FU, follow-up patients; NP, new patient; SD, standard deviation.

[a]A portion of patients will have both NP/EP and subsequent FU visits.

© 2023 Patel KB et al. *JAMA Network Open.*
