# Supplementary material for: Estimated Indirect Cost Savings of Using Telehealth Among Nonelderly Patients With Cancer
Source: JAMA Netw Open. 2023 Jan 10;6(1):e2250211. doi: 10.1001/jamanetworkopen.2022.50211 (PMC9856804; doi:10.1001/jamanetworkopen.2022.50211)
Supplement: Supplement 2. — Data Sharing Statement [file jamanetwopen-e2250211-s002.pdf]

Data Sharing Statement

Patel. Estimated Indirect Cost Savings of Using Telehealth Among Nonelderly Patients With Cancer. *JAMA Netw Open*. Published January 10, 2023.
doi:10.1001/jamanetworkopen.2022.50211

Data
Data available: Yes
Data types: Deidentified participant data
How to access data: krupal.patel@moffitt.org
When available: With publication

Supporting Documents
Document types: None

Additional Information
Who can access the data: Researchers whose proposed use of the data has been approved
Types of analyses: For specified purpose
Mechanisms of data availability: after approval of proposal and with a signed data access agreement
